# Association of D-dimer elevation with inflammation and organ dysfunction in ICU patients with COVID-19 in Wuhan, China: a retrospective observational study

**DOI:** 10.18632/aging.202496

**Published:** 2021-02-11

**Authors:** Wang Zhang, Ling Sang, Jiaran Shi, Ming Zhong, Li Jiang, Bin Song, Liang Kang, Yun Zhang, Dingyu Zhang, Yunsong Yu, Xia Zheng

**Affiliations:** 1 Department of Infectious Diseases, Sir Run Run Shaw Hospital, College of Medicine, Zhejiang University, Hangzhou, Zhejiang, P.R. China; 2 Department of Critical Care Medicine, The First Affiliated Hospital of Guangzhou Medical University, Guangzhou Institute of Respiratory Health, Guangzhou, Guangdong, P.R. China; 3 Department of Cardiology, The First Affiliated Hospital, College of Medicine, Zhejiang University, Hangzhou, Zhejiang, P.R. China; 4 Department of Critical Care Medicine, Zhongshan Hospital, Fudan University, Shanghai, P.R. China; 5 Department of Critical Care Medicine, Xuanwu Hospital, Capital Medical University, Beijing, P.R. China; 6 Department of Tuberculosis and Respiratory Disease, Jinyintan Hospital, Wuhan, Hubei, P.R. China; 7 Department of Critical Care Medicine, Jinyintan Hospital, Wuhan, Hubei, P.R. China; 8 Department of Critical Care Medicine, The First Affiliated Hospital, College of Medicine, Zhejiang University, Hangzhou, Zhejiang, P.R. China; 9 Joint Laboratory of Infectious Diseases and Health, Wuhan Institute of Virology and Wuhan Jinyintan Hospital, Chinese Academy of Sciences, Hubei, P.R. China; 10 Research Center for Translational Medicine, Wuhan Jinyintan Hospital, Hubei, P.R. China

**Keywords:** coronavirus disease 2019, COVID-19, critical care, D-dimer, organ dysfunction, retrospective study

## Abstract

Coronavirus disease 2019 (COVID-19)-associated coagulation dysfunction is gaining attention. In particular, dynamic changes in the D-dimer level may be related to disease progression. Here, we explored whether elevated D-dimer level was related to multiple organ failure and a higher risk of death. This study included 158 patients with COVID-19 who were admitted to the intensive care unit (ICU) at Jinyintan Hospital in Wuhan, China between January 20, 2020 and February 26, 2020. Clinical and laboratory data were collected. The relationship between D-dimer elevation and organ dysfunction was analyzed, as were dynamic changes in inflammation and lipid metabolism. Approximately 63.9% of patients with COVID-19 had an elevated D-dimer level on ICU admission. The 14 day ICU mortality rate was significantly higher in patients with a high D-dimer level than in those with a normal D-dimer level. Patients with a D-dimer level of 10-40μg/mL had similar organ function on ICU admission to those with a D-dimer level of 1.5–10μg/mL. However, patients with higher levels of D-dimer developed organ injuries within 7 days. Furthermore, significant differences in inflammation and lipid metabolism markers were observed between the two groups. In conclusion, the D-dimer level is closely related to COVID-19 severity and might influence the likelihood of rapid onset of organ injury after admission.

## INTRODUCTION

Coronavirus disease 2019 (COVID-19), caused by severe acute respiratory syndrome coronavirus 2, is a considerable threat to public health worldwide. There is increasing evidence that patients with COVID-19 have coagulation dysfunction. In particular, many patients with severe or critical COVID-19 exhibit abnormal coagulation, similar to the systemic coagulopathy associated with severe infection [1], and abnormal coagulation markers are associated with poor prognosis in patients with COVID-19 [2]. Some studies have shown that anticoagulation therapy may improve the prognosis of patients with COVID-19 [3, 4]. However, many such patients (especially those treated in the intensive care unit [ICU]) develop life-threatening thrombotic complications [5].

Thrombotic events frequently occur in patients with COVID-19. Histopathological examination of these patients has revealed a severe pulmonary endothelial injury with widespread thrombosis and microangiopathy, and the rate of the associated alveolar capillary thrombosis is nine fold greater than that of patients with influenza [6]. In addition, multiple histopathological studies have reported thrombosis in extrapulmonary organs, such as the liver and kidneys [7, 8]. There is a need for laboratory and imaging studies to more comprehensively investigate the coagulation complications of COVID-19, including the treatment thereof, and the usefulness of strict thrombosis prophylaxis.

Elevated D-dimer and fibrin degradation product levels, combined with a prolonged prothrombin time, are the most typical finding in patients with COVID-19 and associated coagulopathy [9]. Notably, elevated D-dimer and fibrin degradation product levels are especially common in patients with fatal disease [2]. A study in China showed increasing risk of in-hospital death with a D-dimer elevation [10]. Furthermore, dynamic observation of the D-dimer level has been used to predict the progression of COVID-19 [11]. However, it is unclear whether the D-dimer levels is directly related to multiple organ failure, or whether it is associated with changes in inflammation and metabolism. Here, we assessed the correlations between D-dimer level on ICU admission and hospital mortality, progression of organ injuries, and dynamic changes in inflammation.

## RESULTS

### Demographic and clinical characteristics

In total, 158 patients with severe or critical COVID-19 were included in this study. The median age of the patients was 62 years (interquartile range: 56–70 years) and 91 patients (57.6%) were men. Seventy-seven patients (48.7%) died during treatment in the ICU. Forty-nine patients (31.0%) received anticoagulant therapy and 102 (64.5%) received mechanical ventilation [68 (43.0%) received noninvasive ventilation and 81 (51.3%) received invasive ventilation] (Table 1).

**Table 1:** Characteristics of the study participants with different level of D-dimer.

	**Total**	**D0(n=57)**	**D1(n=38)**	**D2(n=31)**	**D3(n=32)**	***P***
Age	62(56-70)	62(53-70)	61(51-69)	66(63-73)	66(61-69)	**0.046**
Gender, Male	91/158	28/57	18/38	21/31	24/32	**0.035**
Death	77/158	8/57	17/38	22/31	30/32	**<0.001**
Anticoagulant therapy	49/158	14/57	14/28	10/31	11/32	0.593
WBC (×10^9^/L)	10.1(6.4-15.6)	6.5(4.8-9.4)	9.6(6.5-12.5)	12.8(10.2-17.2)^#^	16.2(10.5-19.8)^#^	**<0.001**
Lymphocyte(×10^9^/L)	0.6(0.4-0.9)	0.8(0.6-1.2)	0.5(0.3-0.7)^#^	0.5(0.4-0.7)^#^	0.4(0.3-0.6)^#^	**<0.001**
Neutrophil (×10^9^/L)	8.7(5.0-14.6)	4.9(3.2-8.2)	8.5(5.3-11.9)^*^	11.3(9.2-15.8)^#^	15.2(9.6-19.1)^#^	**<0.001**
Monocyte (×10^9^/L)	0.4(0.2-0.5)	0.4(0.3-0.5)	0.3(0.2-0.4)	0.4(0.3-0.7)	0.4(0.2-0.5)	**0.050**
RBC (×10^12^/L)	3.9(3.5-4.4)	4.0(3.5-4.4)	3.8(3.2-4.5)	3.9(3.5-4.5)	3.8(3.6-4.1)	0.562
HGB (g/L)	116(106-132)	118(108-133)	111(91-134)	117(108-134)	117(108-127)	0.460
PLT (×10^9^/L)	186(144-241)	202(151-241)	236(150-314)	199(179-231)	123(83-174)^#^	**<0.001**
PFDP (μg/mL)	20.2(4.2-81.3)	2.7(1.6-4.2)	12.8(7.6-25.8)^#^	59.0(43.3-81.3)^#^	112.7(100.0-131.7)^#^	**<0.001**
Fbg (g/L)	4.8(3.1-6.1)	4.4(3.6-6.0)	5.1(3.9-5.9)	5.0(2.3-6.3)	4.0(1.9-5.1)	0.082
TT (s)	17.0(16.1-18.6)	17.0(16.2-18.6)	17.1(15.9-18.2)	16.5(16.2-19.3)	17.6(16.8-20.6)	0.334
PT (s)	12.9(11.8-14.7)	11.9(10.9-13.2)	12.4(11.6-13.1)	12.5(11.7-13.7)	14.8(13.9-16.4)^#^	**<0.001**
PTA (%)	79.2(58.7-98.5)	90.8(71.1-108.6)	84.6(72.8-104.2)	81.4(71.7-99.0)	58(50.9-69.3)^#^	**<0.001**
APTT (s)	27.4(23.7-32.2)	28.1(24.4-31.8)	28.0(23.4-34.2)	24.4(20.0-27.0)^*^	30.2(26.0-36.2)	**0.002**
AT3 (%)	89.5(72.8-109.2)	92.2(72.0-119.2)	91.5(76.9-103.4)	93.7(73.6-118.0)	82.1(64.2-100.2)	0.253
CRP (>160 mg/L)	47/154	6/56	8/37	13/31^†^	20/30^†^	**<0.001**
PCT (>0.5 ng/mL)	38/151	5/54	7/37	11/30^†^	15/30^†^	**<0.001**
Serum ferritin (>2000 ng/mL)	45/142	9/54	12/34	11/27	13/27^†^	**0.017**
Cholesterol (mmol/L)	3.4(3.0-4.1)	3.6(3.0-4.2)	3.5(2.9-4.4)	3.5(3.1-3.9)	3.1(2.4-3.7) ^*^	**0.022**
Triglyceride (mmol/L)	1.5(1.1-2.0)	1.2(0.9-1.6)	1.8(1.4-2.3) ^**^	1.4(1.2-1.8)	1.7(1.3-2.3) ^**^	**<0.001**
Apolipoprotein A-1 (g/L)	0.9(0.8-1.0)	1.0(0.8-1.1)	0.9(0.8-1.0)	0.8(0.7-1.0)	0.8(0.6-0.9)^#^	**0.001**
Apolipoprotein B (g/L)	0.8(0.7-1.0)	0.8(0.7-0.9)	0.9(0.7-1.0)	0.9(0.8-1.0)	0.8(0.7-0.9)	0.195
Lipoprotein a (mg/L)	149.5(78.0-273.1)	158.7(100.3-332.5)	149.4(104.8-291.9)	150.1(83.0-272.5)	147.4(64.1-192.9)	0.703
HDL (mmol/L)	0.9(0.7-1.1)	1.0(0.8-1.1)	0.9(0.7-1.1)	0.8(0.7-1.0)	0.6(0.4-0.8) ^#^	**<0.001**
LDL (mmol/L)	1.7(1.5-2.3)	2.0(1.6-2.4)	1.7(1.5-2.4)	2.0(1.7-2.3)	1.5(1.1-2.1) ^*^	**0.022**
hnTNI (pg/mL)	17.0(6.9-75.5)	8.6(3.4-12.7)	22.9(7.0-50.1) ^*^	39.5(9.8-133.8) ^**^	121.2(43.8-846.2) ^#^	**<0.001**
BNP (pg/mL)	51(21-115)	28(11-55)	69(24-119) ^*^	64(33-111) ^*^	131(44-218) ^#^	**<0.001**
LDH (U/L)	453(310-647)	301(230-383)	465(336-627) ^**^	479(419-683) ^#^	714(605-951) ^#^	**<0.001**
HBDH (U/L)	358(235-538)	228(180-305)	394(278-525) ^#^	422(313-605) ^#^	609(485-792) ^#^	**<0.001**
CK (U/L)	105(60-202)	98(62-170)	94(48-178)	119(57-253)	140(75-291)	0.297
CK-MB (U/L)	15(11-22)	12(8-17)	15(9-22)	18(14-24) ^**^	20(16-48) ^#^	**<0.001**
ALB (g/L)	29.5(26.8-31.7)	31.4(29.2-34.8)	30.3(27.9-32.1)	28.0(25.2-29.8) ^#^	27.0(25.0-28.0) ^#^	**<0.001**
ALT (U/L)	38(22-61)	30(15-50)	36(20-56)	42(27-62)	47(31-69)	**0.045**
AST (U/L)	43(31-60)	34(28-49)	43(30-60)	47(33-60)	59(38-72) ^#^	**0.001**
Cholinesterase (U/L)	6308(4773-7659)	6968(5776-8572)	6550(4936-7166)	5511(4216-6309) ^**^	4943(4130-6016) ^#^	**<0.001**
Cr (μmol/L)	73.3(59.7-98.6)	70.8(58.3-83.9)	72.6(58.4-95.6)	73.4(65.5-106.9)	84.5(67.0-108.0)	0.216
eGFR (ml/(min*1.73m^2^))	99.1(70.3-117.6)	104.3(83.6-113.3)	104.8(66.3-130.0)	98.3(62.9-119.2)	87.5(62.2-109.1)	0.401
Uric acid (umol/L)	212(145-299)	235(188-319)	176(136-276)	216(138-303)	241(124-288)	0.199
Cystatin-c (mg/L)	1.1(0.9-1.4)	1.0(0.8-1.1)	1.3(1.0-1.5) ^**^	1.1(0.9-1.3)	1.2(1.0-1.6) ^**^	**0.001**
DIC (ISTH criteria)	21/158	0/57	6/38^†^	6/31^†^	9/32^†^	**<0.001**
PaO_2_/FiO_2_<100	74/158	23/57	23/38	14/31	14/32	0.264
SOFA score	4(3-5)	3(2-4)	4(3-5)^*^	5(4-5)^**^	6(4-7) ^#^	**<0.001**
NIV	68/158	10/57	17/38	16/31	25/32	**<0.001**
IMV	81/158	10/57	24/38	25/31	22/32	**<0.001**
NIV+IMV	102/158	16/57	27/38	28/31	31/32	**<0.001**

Compared with D0 group, *<0.05; **<0.01; #<0.001. † means P<0.05 after adjusting by Bonferroni way.

### D-dimer level on ICU admission was correlated with laboratory test results

**Figure 1:**
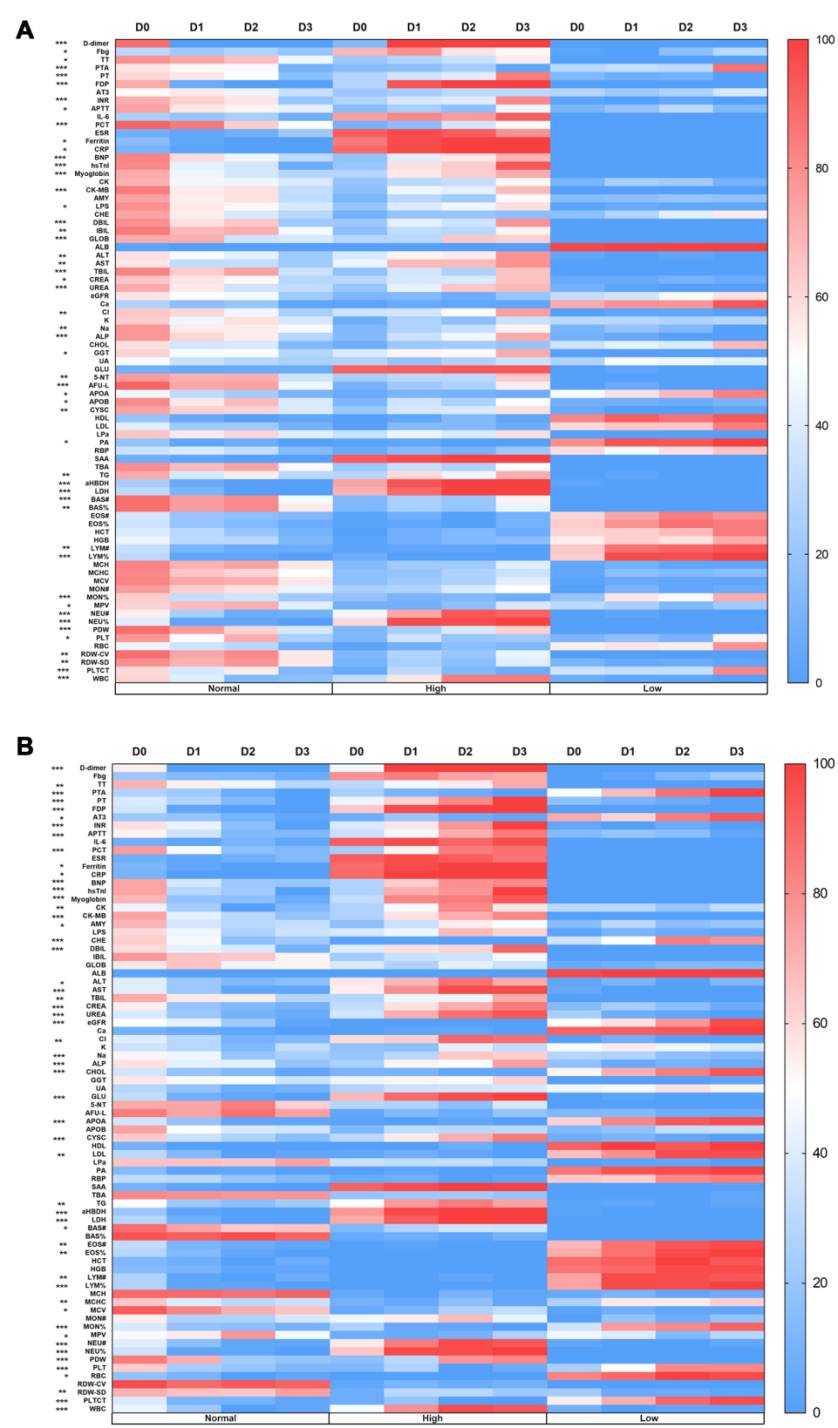
**Heat maps of the correlation between the D-dimer level and laboratory results.** (**A**) The number of patients with abnormalities in 46 parameters varied according to D-dimer level at the time of ICU admission. (**B**) The number of patients with abnormalities in 52 parameters varied according to D-dimer level during ICU treatment. D0: D-dimer<1.5μg/mL, D1: 1.5≤D-dimer<10μg/mL, D2: 10≤D-dimer<40μg/mL, D3: D-dimer≥40μg/mL. Normal: the result is within the normal range, High: the result is beyond the normal range, Low: the result is below the normal range. *P<0.05, **P<0.01, ***P<0.001 among these 4 groups, abbreviation and range of normal values can be found in Supplementary Table 3, Supplementary files.

The 158 patients were divided into four groups based on their D-dimer level at the time of ICU admission. The D0 group comprised patients with a normal D-dimer level (D-dimer<1.5μg/mL). The D1–D3 groups comprised patients with an abnormal D-dimer level, as follows: D1 (1.5≤D-dimer<10μg/mL), D2 (10≤D-dimer<40μg/mL) and D3 (D-dimer≥ 40μg/mL). The laboratory test results were divided into three categories (abbreviations and ranges of normal values are shown in Supplementary Table 3): high (above normal range), normal (within normal range), and low (below normal range). Heat maps were used to show the numbers of patients in the various laboratory test result categories by D-dimer level (Figure 1). At the time of ICU admission, the number of patients with abnormalities in 46 parameters varied by D-dimer level (Figure 1A). During ICU treatment, the number of patients with abnormalities in 52 parameters varied by D-dimer level (Figure 1B). These parameters reflected coagulation function, inflammation level, liver function, renal function, lipid metabolism and myocardial injury. Furthermore, the values of these parameters significantly differed among the four D-dimer groups (Table 1). We evaluated the correlations between D-dimer level and other laboratory test results. The D-dimer level was positively correlated with the white blood cell count (WBC, r = 0.523, P < 0.001), neutrophil count (r = 0.557, P < 0.001), CRP level (r = 0.441, P < 0.001), procalcitonin level (r = 0.357, P < 0.001), hypersensitive sensitive troponin I level (r = 0.562, P < 0.001), lactate dehydrogenase level (r = 0.633, P < 0.001) and α-Hydroxybutyrate dehydrogenase level (r = 0.654, P < 0.001) for all 158 patients on the first day after ICU admission. On that day, the D-dimer level was negatively correlated with the lymphocyte count (r = -0.419, P < 0.001) and albumin level (r = -0.524, P < 0.001) (Supplementary Table 1). The results of multivariate Cox regression analysis showed increasing odds of in-ICU death with older age, a higher SOFA score, and higher D-dimer, lactate dehydrogenase, apolipoprotein A, and apolipoprotein B levels on ICU admission (Supplementary Table 2).

### D-dimer level on ICU admission was associated with 7- and 14-day mortality rates

**Figure 2:**
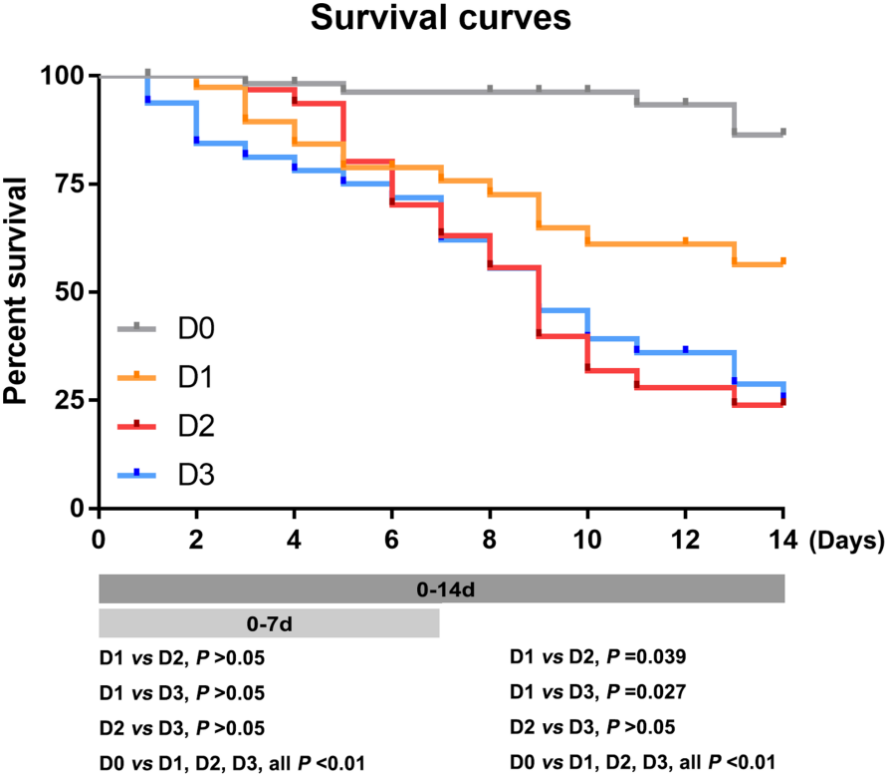
**The 7- and 14-day mortality rates in patients with different D-dimer levels at ICU admission.** Survival curves of patients with different D-dimer level within 7 and 14 days. D0: D-dimer<1.5μg/mL, D1: 1.5≤D-dimer<10μg/mL, D2: 10≤D-dimer<40μg/mL, D3: D-dimer≥40μg/mL.

There were no significant differences in the 7-day mortality rate among the D1, D2, and D3 groups. Within 14 days after ICU admission, the death rates were as follows: D0 group, 5 of 57 patients, 13.66%; D1 group, 14 of 38 patients, 43.57%; D2 group, 21 of 31 patients, 76.12%; and D3 group, 23 of 32 patients,74.84% (Figure 2, Supplementary Figure 2). The 14-day mortality rate was significantly lower in the D0 group than the D1, D2, and D3 groups (all P<0.01), and significantly lower in the D1 group than in the D2 and D3 groups (all P<0.05). However, there was no significant difference in the 14 day mortality rate between the D2 and D3 groups (p=0.963) (Figure 2). In addition, there was no significant difference in the ICU mortality rate between male and female patients (Supplementary Figure 1).

### D-dimer level on ICU admission was associated with dynamic changes in organ function during ICU treatment

**Figure 3:**
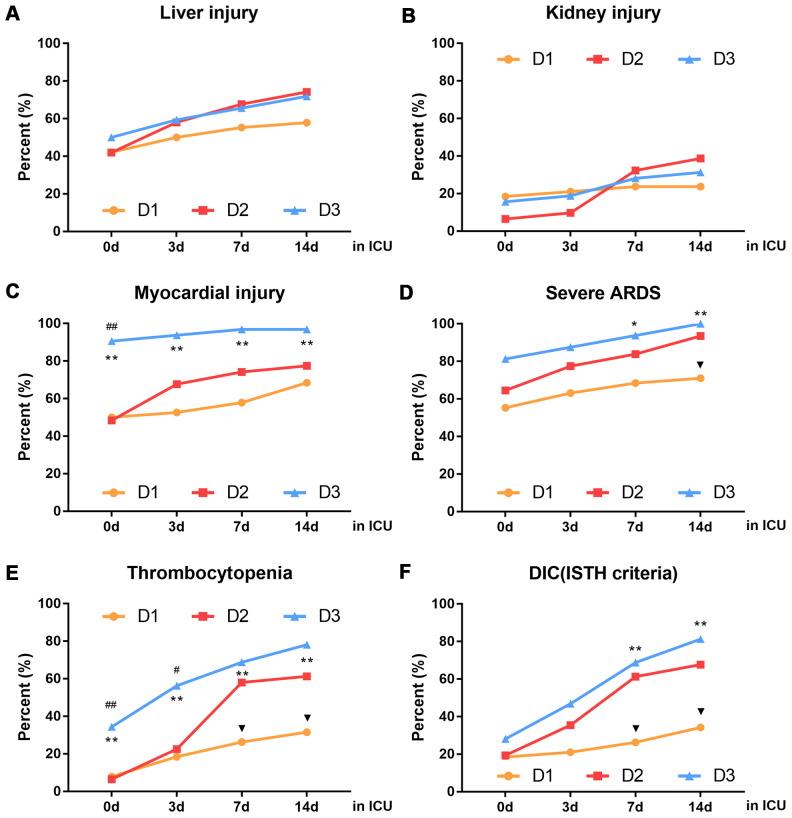
**Dynamic changes in organ function in patients with different D-dimer levels within 14 days after ICU admission.** (**A**) liver injury (**B**) kidney injury (**C**) myocardial injury (**D**) severe ARDS (**E**) thrombocytopenia (**F**) DIC (ISTH criteria), D1: 1.5≤D-dimer<10μg/mL, D2: 10≤D-dimer<40μg/mL, D3: D-dimer≥40μg/mL. ^▼^P<0.05 with D1 group vs. D2 group, ^#^P<0.05, ^##^P<0.01 with D2 group vs. D3 group, *P<0.05, **P<0.01 with D1 group vs. D3 group. ARDS: acute respiratory distress syndrome, DIC: disseminated intravascular coagulation, ISTH: international society of thrombosis and hemostasis.

Organ dysfunction was related to the D-dimer level at the time of ICU admission: in the D3 group, 16 of 32 patients (50%) had abnormal liver function, 5 of 32 (15.63%) had abnormal renal function, 29 of 32 (90.63%) had myocardial injury, 11 of 32(34.38%) had thrombocytopenia. 26 of 32 (81.25%) had severe ARDS and 9 of 32 (28.13%) had DIC. There was no significant difference in organ function status between the D1 and D2 groups at the time of ICU admission (Figure 3). However, patients in the D2 group experienced rapid deterioration of organ function after ICU admission (Figure 3C–3F). In particular, significantly more patients in the D2 group had thrombocytopenia and DIC, compared with the D1 group, within 4-7 days after ICU admission (Figure 3E, 3F). Additionally, significantly more patients in the D2 group had severe ARDS, compared with the D1 group, within 8-14 days after ICU admission (Figure 3D). Notably, by day 14 after ICU admission, the numbers of patients with liver injury, kidney injury, severe ARDS and DIC were similar between the D2 and D3 groups (Figure 3A, 3B, 3D–3F).

**Figure 4:**
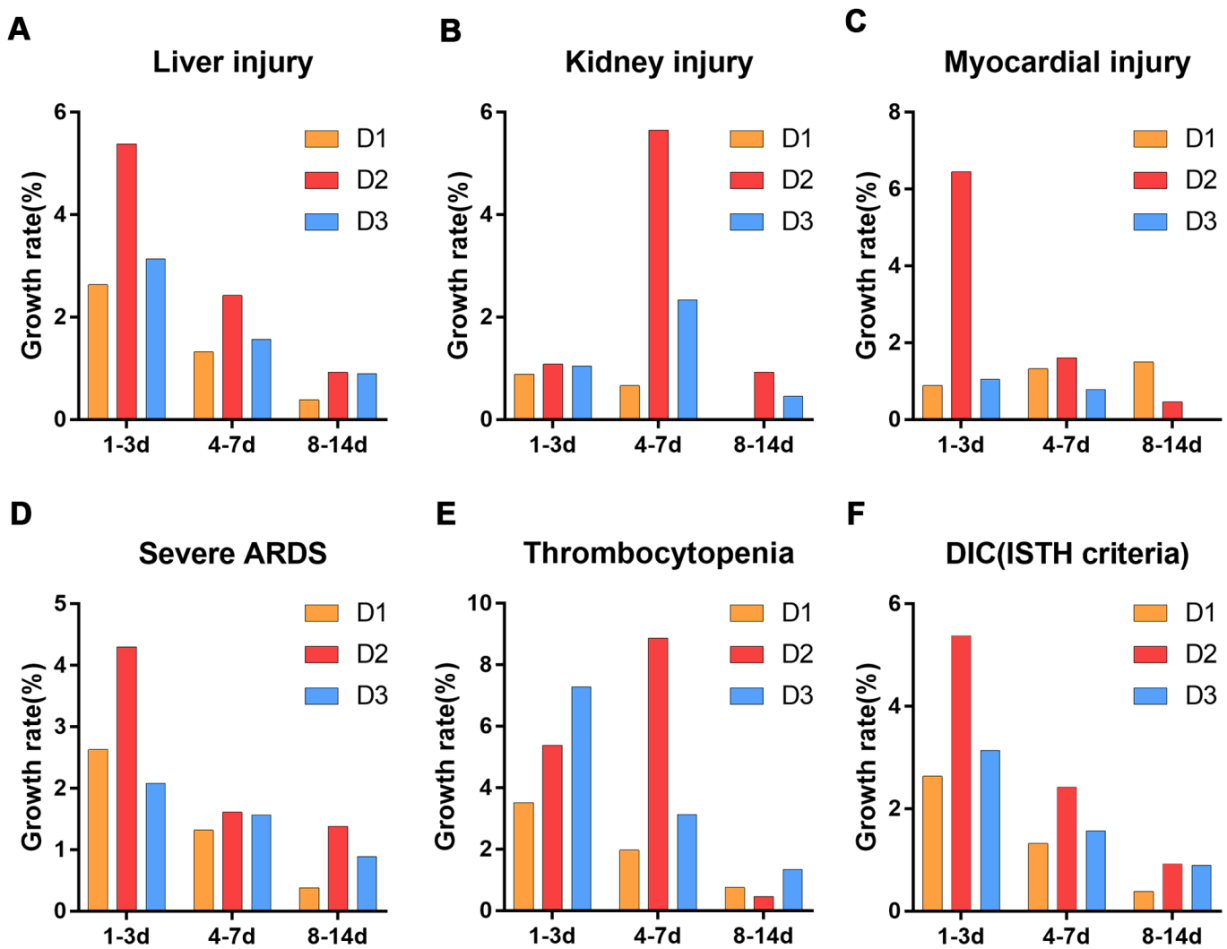
**Growth rate of the number of patients with organ dysfunction after ICU admission.** (**A**) liver injury (**B**) kidney injury (**C**) myocardial injury (**D**) severe ARDS (**E**) thrombocytopenia (**F**) DIC (ISTH criteria). D1: 1.5≤D-dimer<10μg/mL, D2: 10≤D-dimer<40μg/mL, D3: D-dimer≥40μg/mL. ARDS: acute respiratory distress syndrome, DIC: disseminated intravascular coagulation, ISTH: international society of thrombosis and hemostasis.

Further analysis of the numbers of patients with organ dysfunction over time showed that the growth rates of the numbers of patients with liver injury, myocardial injury, severe ARDS and DIC peaked at 1-3 days after ICU admission (Figure 4A, 4C–4F), while the growth rates of the numbers of patients with kidney injury and thrombocytopenia peaked at 4-7 days after ICU admission (Figure 4B, 4E). Importantly, within 7 days after ICU admission, the growth rates of the numbers of patients with organ dysfunction was higher in the D2 group than in the other three groups (Figure 4).

### Dynamic relationships of inflammation and lipid metabolism markers with D-dimer level

**Figure 5:**
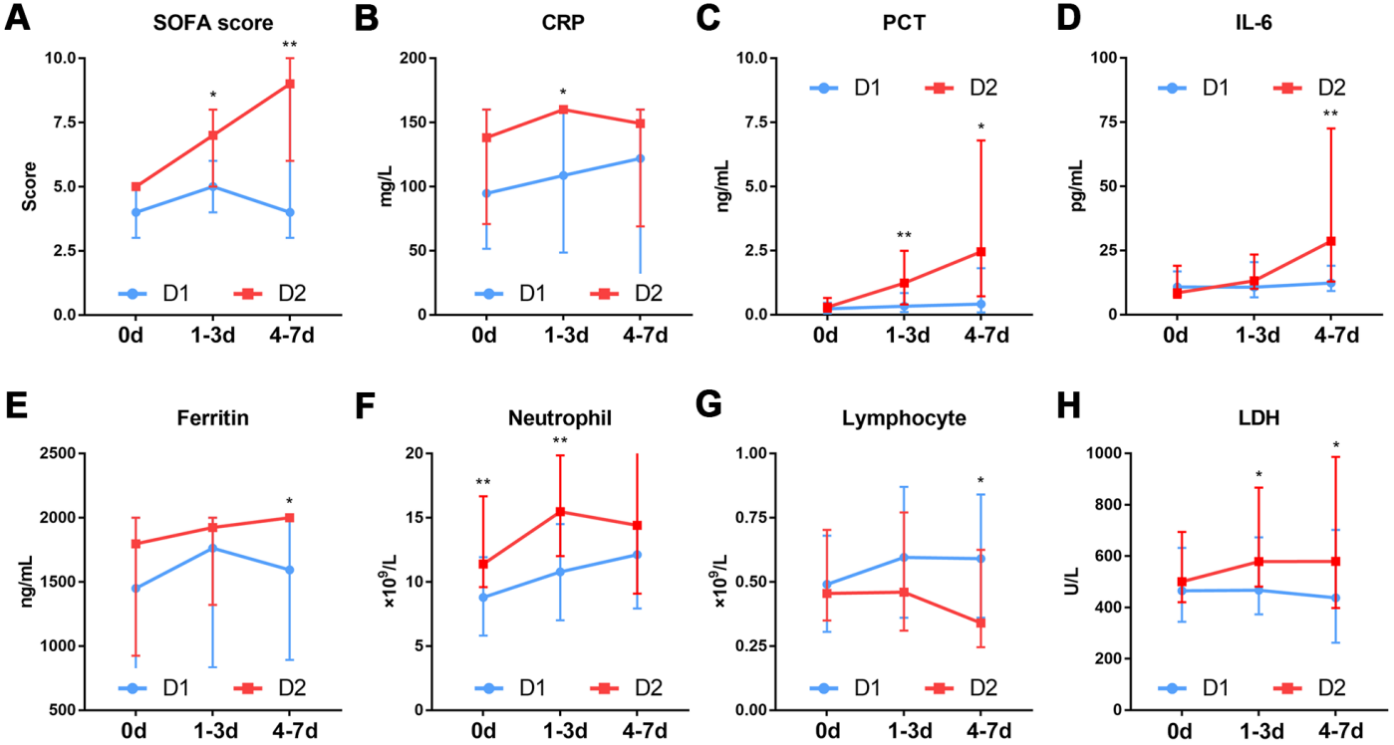
**Dynamic changes in the SOFA score and inflammation markers in patients with different D-dimer levels.** (**A**) SOFA score (**B**) CRP (**C**) PCT (**D**) IL-6 (**E**) Ferritin (**F**) Neutrophil. (**G**)Lymphocyte (**H**) LDH. D1: 1.5≤D-dimer<10μg/mL, D2: 10≤D-dimer<40μg/mL. SOFA: sequential organ failure assessment, CRP: c-reactive protein, PCT: procalcitonin, IL-6: Interleukin-6, LDH: Lactate dehydrogenase. *P<0.05, **P<0.01 with D1 group vs. D2 group.

**Figure 6:**
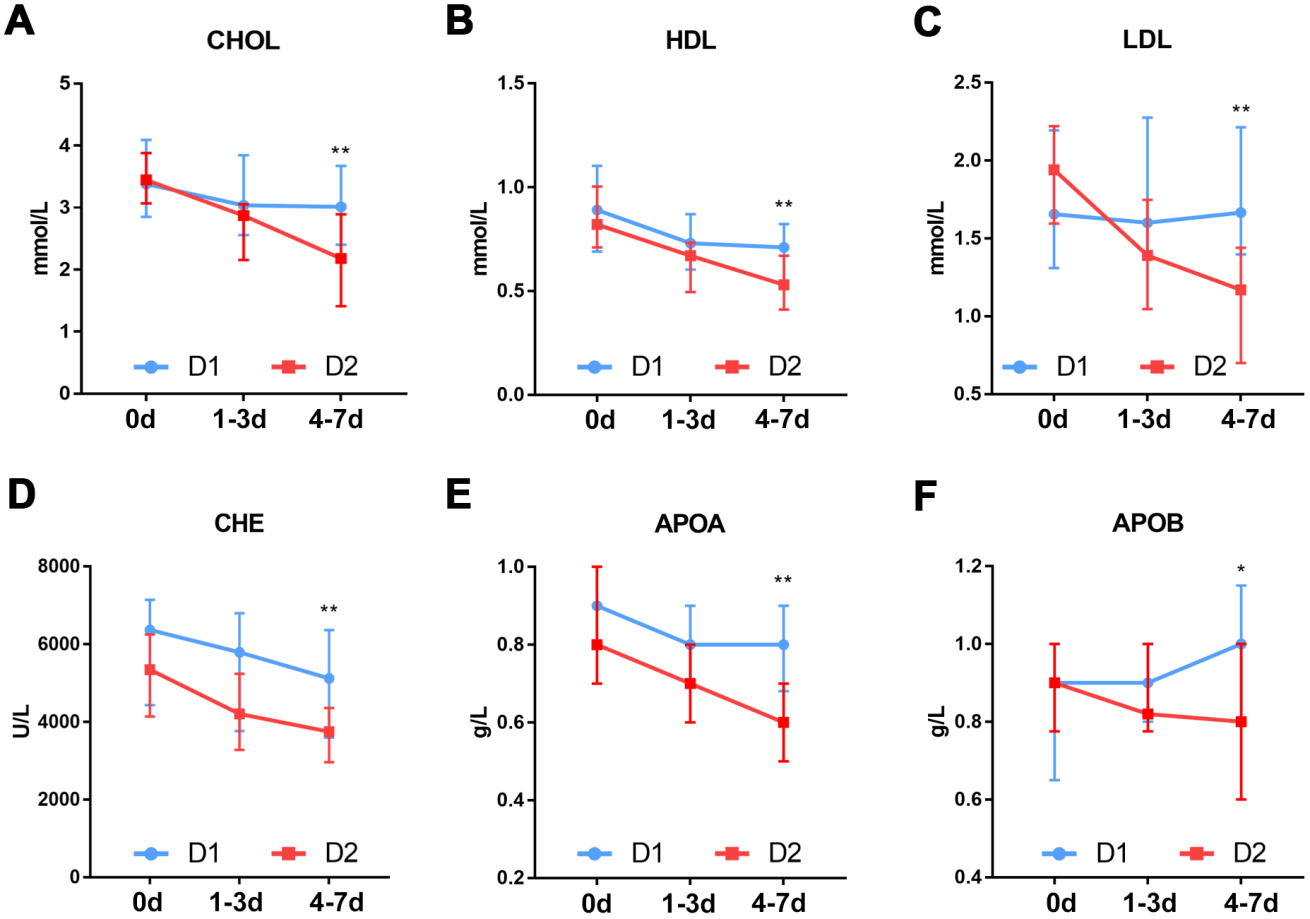
**Dynamic changes in lipid metabolism markers in patients with different D-dimer levels.** (**A**) CHOL (**B**) HDL (**C**) LDL (**D**) CHE (**E**) APOA (**F**) APOB. D1: 1.5≤D-dimer<10μg/mL, D2: 10≤D-dimer<40μg/mL. CHOL: Cholesterol, HDL: High density lipoprotein, LDL: Low density lipoprotein, CHE: Cholinesterase, APOA: Apolipoprotein A, APOB: Apolipoprotein B. *P<0.05, **P<0.01 with D1 group vs. D2 group.

To investigate the causes of rapid progression of organ dysfunction in the patients in the D2 group, we compared the dynamic changes in SOFA score, inflammation status and whole blood cells between the D1 and D2 group patients within 7 days after ICU admission. The results showed that the SOFA score was significantly higher in patients in the D2 group than in those in the D1 group, both 1-3 and 4-7days after ICU admission (Figure 5A). With the exception of neutrophil count (P=0.003), there was no significant difference in any other parameter between the two groups, at the time of ICU admission (Figure 5B–5H). At 1-3 days after ICU admission, the levels of inflammation-related markers such as CRP (P=0.018), procalcitonin (P=0.004) and lactate dehydrogenase (P=0.026), were significantly different between the two groups (Figure 5B–5H). At 4-7 days after ICU admission, there were significant differences between the two groups in the levels of inflammation-related markers such as lymphocyte count (P=0.029), IL-6 (P=0.008) and ferritin (P=0.026) (Figure 5D–5G), as well as in the levels of lipid metabolism-related markers such as lipoprotein (low-density lipoprotein, P=0.031; high-density lipoprotein, P=0.001), cholinesterase (P=0.007), cholesterol (P=0.005), apolipoprotein (apolipoprotein A, P=0.001; apolipoprotein B, P=0.035) (Figure 6).

## DISCUSSION

Coagulation dysfunction is common in critically ill patients with COVID-19 [2]. Autopsy results have shown thrombosis in the lungs of patients with COVID-19, as well as in the heart, liver and kidneys [6, 7, 12, 13]. Notably, some patients develop thrombosis despite preventive anticoagulation therapy [8]. COVID-19-related coagulation dysfunction is distinct from DIC and thrombotic microangiopathy; it is a unique syndrome [14]. Increasing levels of inflammatory factors in the circulation, as well as the activation of a large number of platelets and the destruction of vascular endothelial cells lead to blood stasis and hypercoagulability. Together, these effects cause extensive intravascular and microvascular thrombosis [15]. Accordingly, secondary fibrin dissolution is enhanced, which ultimately leads to elevated levels of fibrin degradation products such as D-dimer [16]. From this perspective, the elevated D-dimer level in patients with COVID-19 is non-specific. However, considering the extensive intravascular thrombosis and DIC in these patients, an elevated D-dimer level may be informative regarding disease assessment, clinical diagnosis, and treatment selection.

An elevated D-dimer level at admission was associated with higher hospital mortality in patients with COVID-19 in Wuhan [10]. In our study, because of the large number of critically ill patients in the early stage of the epidemic, some of the patients had an extremely high D-dimer level. In particular, 39.9% of the patients had a D-dimer level> 10μg/mL, while 20% had a D-dimer level> 40μg/mL. Notably, few studies have included patients with such high levels of D-dimer. An autopsy study of patients with COVID-19 from China, including those who underwent systematic autopsy (n=37 patients) and those who underwent percutaneous multiple organ biopsy (i.e., “minimally invasive autopsy”, n= 54), revealed that the main cause of COVID-19-related death was multiple organ dysfunction syndrome, especially ARDS. Infection with severe acute respiratory syndrome coronavirus 2 can cause varying degrees of acute injury to multiple organs. This results in multiple organ dysfunction syndrome, in which lesions within the lung, heart, kidney and liver have serious consequences [17]. Further analysis showed that patients with D-dimer levels of 1.5-10 μg/mL and those with D-dimer levels of 10-40 μg/mL had similar disease severity (in terms of liver injury, kidney injury, myocardial injury, severe ARDS, thrombocytopenia, and DIC). Moreover, survival analysis revealed no difference in 7-day mortality rate between the two groups. However, the 14-day mortality rate was significantly higher in patients with a D-dimer level of 10–40 μg/mL than in those with a level of 1.5-10 μg/ml. These findings imply that the patients with a D-dimer level of 10-40 μg/mL may experience rapid progression of disease with organ injury as the main manifestation 7 days after ICU admission.

There is increasing evidence that severe COVID-19 reflects a confluence of vascular dysfunction, coagulation dysfunction, and dysregulated inflammation [18]. Our analysis of patients with D-dimer levels of 10-40 and 1.5-10μg/ml revealed differences in the levels of inflammation markers (e.g., CRP, procalcitonin and lactate dehydrogenase) at 1-3 days after ICU admission, whereas the IL-6 level, ferritin level and lymphocyte count were differed at 4-7 days after ICU admission. The association between coagulation and inflammation is presumed to result from crosstalk [19, 20]. During an inflammatory reaction, the secreted inflammatory mediators trigger blood coagulation and may cause blood coagulation disorders [21]. Some key components of the coagulation system can promote inflammation by direct and indirect mechanisms, according to their effects on tissue factor and fibrinogen, for example. These components of the coagulation process also have multiple roles in tissue damage and inflammation [20, 22]. For patients with a D-dimer level of 10-40 μg/ml, early organ protection and anti-inflammatory treatment within 4-7 days after ICU admission may be beneficial. In addition, our multivariate Cox regression analysis showed the that lipids such as apolipoprotein A and B were independent risk factors for death. A recent proteomic and metabolomic profiling analysis of serum samples from patients with COVID-19 revealed that more than 100 types of amino acids and lipids were significantly reduced in serum samples from patients with severe COVID-19, which was presumably related to their consumption during rapid viral replication [23]. Similarly, we found significant differences in lipids and their metabolites between our D1 and D2 groups at 4-7 days after ICU admission. Changes in lipid metabolism also reflect changes in systemic inflammation in critically ill patients [24–26]. Therefore, the progression of organ injuries in patients with a D-dimer level of 10-40 μg/ml within 4-7 days after ICU admission may be related to a worsening of the inflammatory state during this period. As noted above, early organ protection and anti-inflammatory treatment may be beneficial to these patients. However, these findings should be confirmed in independent clinical studies. Our results revealed that the D-dimer level can be used to predict the prognosis of critically ill patients with COVID-19, to promote rational allocation of medical resources, and provide some guidance for the selection of drugs for these critically ill patients. Importantly, early intervention for these patients may prevent organ dysfunction.

## CONCLUSIONS

An elevated D-dimer level is common among critically ill ICU patients with COVID-19. Elevated D-dimer levels, especially to 10-40 μg/mL, are associated with a higher 14-day mortality rate in the ICU, and with the occurrence and progression of organ dysfunction in critically ill patients with COVID-19. Notably, this may be related to the secondary inflammatory state that occurs within 4-7 days after ICU admission.

## MATERIALS AND METHODS

### Study design and data collection

This study included 158 patients (≥18 years of age) with confirmed COVID-19 who were admitted to ICUs in Jinyintan Hospital (a designated hospital for patients with COVID-19 in Wuhan, China) between January 20, 2020, and February 26, 2020. All patients were diagnosed with COVID-19 (severe and critical type) in accordance with the World Health Organization interim guidance and Chinese guidelines for COVID-19 management (version 7.0) [27, 28]. Epidemiological data, clinical findings, laboratory test results, and clinical records were obtained from the electronic medical record system. All data were collected by two researchers and then reviewed and confirmed by the authors of this study. The primary clinical outcome, all-cause death was monitored until March 4, 2020 (i.e., the final follow-up). This study was approved by the Research Ethics Commission of Jinyintan Hospital (KY-2020–56.01).

### Laboratory procedures

The clinical laboratory of Jinyintan Hospital detected severe acute respiratory syndrome coronavirus 2 nucleic acid using the real-time reverse transcriptase polymerase chain reaction assay as described previously [29]. All laboratory data collected within 14 days after ICU admission were included in the analysis. Laboratory analyses included complete blood count, coagulation function parameters (e.g., D-dimer, fibrinogen, prothrombin time, prothrombin activity, thromboplastin time, Activated partial thromboplastin time, fibrin degradation products, and antithrombin III, inflammation markers (e.g., interleukin [IL]-6, serum ferritin, C-reactive protein [CRP], procalcitonin, and lactate dehydrogenase), and various other assessments (liver function, renal function, electrolytes and myocardial enzymes). All clinical laboratory data were generated by the clinical laboratory of Jinyintan Hospital.

### Definition

COVID-19 severity was defined on the basis of the Chinese guidelines for COVID-19 management (version 7.0) [28]. Disseminated intravascular coagulation (DIC) was defined in accordance with the criteria established by the International Society of Thrombosis and Hemostasis [30]. The Berlin definition of acute respiratory distress syndrome (ARDS) was used [31]. Acute kidney injury, liver injury and cardiac injury were defined in accordance with a previous study [32].

### Statistical analysis

Continuous data were compared using the Mann–Whitney U-test or Kruskal–Wallis test and are presented as the median (interquartile range). Categorical data were compared using the chi-square test or Fisher’s exact test, and are presented as counts. Univariate Cox regression analysis was used to screen potential predictors. Considering collinearity between variables, multivariate regression ultimately included age, gender, the Sequential Organ Failure Assessment (SOFA) score, D-dimer level, hypersensitive sensitive troponin I, white blood cell count, and levels of lymphocytes, C-reactive protein, lactate dehydrogenase, apolipoprotein A, and apolipoprotein B. Spearman rank correlation analysis was used to explore the association between D-dimer subgroup and clinical characteristics. Kaplan–Meier analysis was used to evaluate the cumulative mortality of patients with different D-dimer levels in the ICU. SPSS 23.0 and GraphPad Prism 7.0 were used for the analyses. P<0.05 was considered statistically significant.

## Supplementary Material

Supplementary Figures

Supplementary Tables

## References

[1] Bhatraju PK, Ghassemieh BJ, Nichols M, Kim R, Jerome KR, Nalla AK, Greninger AL, Pipavath S, Wurfel MM, Evans L, Kritek PA, West TE, Luks A, et al. Covid-19 in critically ill patients in the Seattle region - case series. N Engl J Med. 2020; 382:2012–22. 10.1056/NEJMoa2004500 32227758 PMC7143164

[2] Tang N, Li D, Wang X, Sun Z. Abnormal coagulation parameters are associated with poor prognosis in patients with novel coronavirus pneumonia. J Thromb Haemost. 2020; 18:844–47. 10.1111/jth.14768 32073213 PMC7166509

[3] Li T, Lu H, Zhang W. Clinical observation and management of COVID-19 patients. Emerg Microbes Infect. 2020; 9:687–90. 10.1080/22221751.2020.1741327 32208840 PMC7103696

[4] Tang N, Bai H, Chen X, Gong J, Li D, Sun Z. Anticoagulant treatment is associated with decreased mortality in severe coronavirus disease 2019 patients with coagulopathy. J Thromb Haemost. 2020; 18:1094–99. 10.1111/jth.14817 32220112 PMC9906401

[5] Helms J, Tacquard C, Severac F, Leonard-Lorant I, Ohana M, Delabranche X, Merdji H, Clere-Jehl R, Schenck M, Fagot Gandet F, Fafi-Kremer S, Castelain V, Schneider F, et al, and CRICS TRIGGERSEP Group (Clinical Research in Intensive Care and Sepsis Trial Group for Global Evaluation and Research in Sepsis). High risk of thrombosis in patients with severe SARS-CoV-2 infection: a multicenter prospective cohort study. Intensive Care Med. 2020; 46:1089–98. 10.1007/s00134-020-06062-x 32367170 PMC7197634

[6] Ackermann M, Verleden SE, Kuehnel M, Haverich A, Welte T, Laenger F, Vanstapel A, Werlein C, Stark H, Tzankov A, Li WW, Li VW, Mentzer SJ, Jonigk D. Pulmonary vascular endothelialitis, thrombosis, and angiogenesis in covid-19. N Engl J Med. 2020; 383:120–28. 10.1056/NEJMoa2015432 32437596 PMC7412750

[7] Wichmann D, Sperhake JP, Lütgehetmann M, Steurer S, Edler C, Heinemann A, Heinrich F, Mushumba H, Kniep I, Schröder AS, Burdelski C, de Heer G, Nierhaus A, et al. Autopsy findings and venous thromboembolism in patients with COVID-19: a prospective cohort study. Ann Intern Med. 2020; 173:268–77. 10.7326/M20-2003 32374815 PMC7240772

[8] Lax SF, Skok K, Zechner P, Kessler HH, Kaufmann N, Koelblinger C, Vander K, Bargfrieder U, Trauner M. Pulmonary arterial thrombosis in COVID-19 with fatal outcome : results from a prospective, single-center, clinicopathologic case series. Ann Intern Med. 2020; 173:350–61. 10.7326/M20-2566 32422076 PMC7249507

[9] Levi M, Thachil J, Iba T, Levy JH. Coagulation abnormalities and thrombosis in patients with COVID-19. Lancet Haematol. 2020; 7:e438–40. 10.1016/S2352-3026(20)30145-9 32407672 PMC7213964

[10] Zhou F, Yu T, Du R, Fan G, Liu Y, Liu Z, Xiang J, Wang Y, Song B, Gu X, Guan L, Wei Y, Li H, et al. Clinical course and risk factors for mortality of adult inpatients with COVID-19 in Wuhan, China: a retrospective cohort study. Lancet. 2020; 395:1054–62. 10.1016/S0140-6736(20)30566-3 32171076 PMC7270627

[11] Li Y, Zhao K, Wei H, Chen W, Wang W, Jia L, Liu Q, Zhang J, Shan T, Peng Z, Liu Y, Yan X. Dynamic relationship between D-dimer and COVID-19 severity. Br J Haematol. 2020; 190:e24–27. 10.1111/bjh.16811 32420615 PMC7276819

[12] Harenberg J, Favaloro E. COVID-19: progression of disease and intravascular coagulation - present status and future perspectives. Clin Chem Lab Med. 2020; 58:1029–36. 10.1515/cclm-2020-0502 32406381

[13] Menter T, Haslbauer JD, Nienhold R, Savic S, Hopfer H, Deigendesch N, Frank S, Turek D, Willi N, Pargger H, Bassetti S, Leuppi JD, Cathomas G, et al. Postmortem examination of COVID-19 patients reveals diffuse alveolar damage with severe capillary congestion and variegated findings in lungs and other organs suggesting vascular dysfunction. Histopathology. 2020; 77:198–209. 10.1111/his.14134 32364264 PMC7496150

[14] Giannis D, Ziogas IA, Gianni P. Coagulation disorders in coronavirus infected patients: COVID-19, SARS-CoV-1, MERS-CoV and lessons from the past. J Clin Virol. 2020; 127:104362. 10.1016/j.jcv.2020.104362 32305883 PMC7195278

[15] Wang J, Saguner AM, An J, Ning Y, Yan Y, Li G. Dysfunctional coagulation in COVID-19: from cell to bedside. Adv Ther. 2020; 37:3033–39. 10.1007/s12325-020-01399-7 32504450 PMC7274265

[16] Lippi G, Favaloro EJ. D-dimer is associated with severity of coronavirus disease 2019: a pooled analysis. Thromb Haemost. 2020; 120:876–78. 10.1055/s-0040-1709650 32246450 PMC7295300

[17] Bian XW, and The COVID-19 Pathology Team. Autopsy of COVID-19 victims in China. National Science Review. 2020; 7:1414–18. 10.1093/nsr/nwaa123 PMC7313767 34192086

[18] Leisman DE, Deutschman CS, Legrand M. Facing COVID-19 in the ICU: vascular dysfunction, thrombosis, and dysregulated inflammation. Intensive Care Med. 2020; 46:1105–08. 10.1007/s00134-020-06059-6 32347323 PMC7186535

[19] Burzynski LC, Clarke MC. Death is coming and the Clot thickens, as pyroptosis feeds the fire. Immunity. 2019; 50:1339–41. 10.1016/j.immuni.2019.05.015 31216455

[20] Luyendyk JP, Schoenecker JG, Flick MJ. The multifaceted role of fibrinogen in tissue injury and inflammation. Blood. 2019; 133:511–20. 10.1182/blood-2018-07-818211 30523120 PMC6367649

[21] Marshall JC. Inflammation, coagulopathy, and the pathogenesis of multiple organ dysfunction syndrome. Crit Care Med. 2001; 29:S99–106. 10.1097/00003246-200107001-00032 11445742

[22] Mackman N. The many faces of tissue factor. J Thromb Haemost. 2009 (Suppl 1); 7:136–39. 10.1111/j.1538-7836.2009.03368.x 19630786 PMC2834482

[23] Shen B, Yi X, Sun Y, Bi X, Du J, Zhang C, Quan S, Zhang F, Sun R, Qian L, Ge W, Liu W, Liang S, et al. Proteomic and metabolomic characterization of COVID-19 patient sera. Cell. 2020; 182:59–72.e15. 10.1016/j.cell.2020.05.032 32492406 PMC7254001

[24] Golucci AP, Marson FA, Ribeiro AF, Nogueira RJ. Lipid profile associated with the systemic inflammatory response syndrome and sepsis in critically ill patients. Nutrition. 2018; 55:7–14. 10.1016/j.nut.2018.04.007 29960160

[25] Lekkou A, Mouzaki A, Siagris D, Ravani I, Gogos CA. Serum lipid profile, cytokine production, and clinical outcome in patients with severe sepsis. J Crit Care. 2014; 29:723–27. 10.1016/j.jcrc.2014.04.018 24891152

[26] Ilias I, Vassiliadi DA, Theodorakopoulou M, Boutati E, Maratou E, Mitrou P, Nikitas N, Apollonatou S, Dimitriadis G, Armaganidis A, Dimopoulou I. Adipose tissue lipolysis and circulating lipids in acute and subacute critical illness: effects of shock and treatment. J Crit Care. 2014; 29:1130.e5–9. 10.1016/j.jcrc.2014.06.003 25012960

[27] WHO. Clinical management of severe acute respiratory infection when Novel coronavirus (nCoV) infection is suspected: interim guidance, 2020 Jan 28. 2020.

[28] National Health Commission of the People’s Republic of China. Chinese management guideline for COVID-19 (version 7.0). 2020 Feb 19, 2020. http://www.nhc.gov.cn/yzygj/s7653p/202002/8334a8326dd94d329df351d7da8aefc2/files/b218cfeb1bc54639af227f922bf6b817.pdf

[29] Huang C, Wang Y, Li X, Ren L, Zhao J, Hu Y, Zhang L, Fan G, Xu J, Gu X, Cheng Z, Yu T, Xia J, et al. Clinical features of patients infected with 2019 novel coronavirus in Wuhan, China. Lancet. 2020; 395:497–506. 10.1016/S0140-6736(20)30183-5 31986264 PMC7159299

[30] Gando S, Levi M, Toh CH. Disseminated intravascular coagulation. Nat Rev Dis Primers. 2016; 2:16037. 10.1038/nrdp.2016.37 27250996

[31] Ranieri VM, Rubenfeld GD, Thompson BT, Ferguson ND, Caldwell E, Fan E, Camporota L, Slutsky AS, and ARDS Definition Task Force. Acute respiratory distress syndrome: the berlin definition. JAMA. 2012; 307:2526–33. 10.1001/jama.2012.5669 22797452

[32] Wang D, Hu B, Hu C, Zhu F, Liu X, Zhang J, Wang B, Xiang H, Cheng Z, Xiong Y, Zhao Y, Li Y, Wang X, Peng Z. Clinical characteristics of 138 Hospitalized patients with 2019 novel coronavirus-infected pneumonia in Wuhan, China. JAMA. 2020; 323:1061–69. 10.1001/jama.2020.1585 32031570 PMC7042881

