# Supplementary material for: Association of D-dimer elevation with inflammation and organ dysfunction in ICU patients with COVID-19 in Wuhan, China: a retrospective observational study
Source: Aging (Albany NY). 2021 Feb 11;13(4):4794–810. doi: 10.18632/aging.202496 (PMC7950237; doi:10.18632/aging.202496)
Supplement: Supplementary Figures [file aging-13-202496-s001.pdf]

SUPPLEMENTARY FIGURES

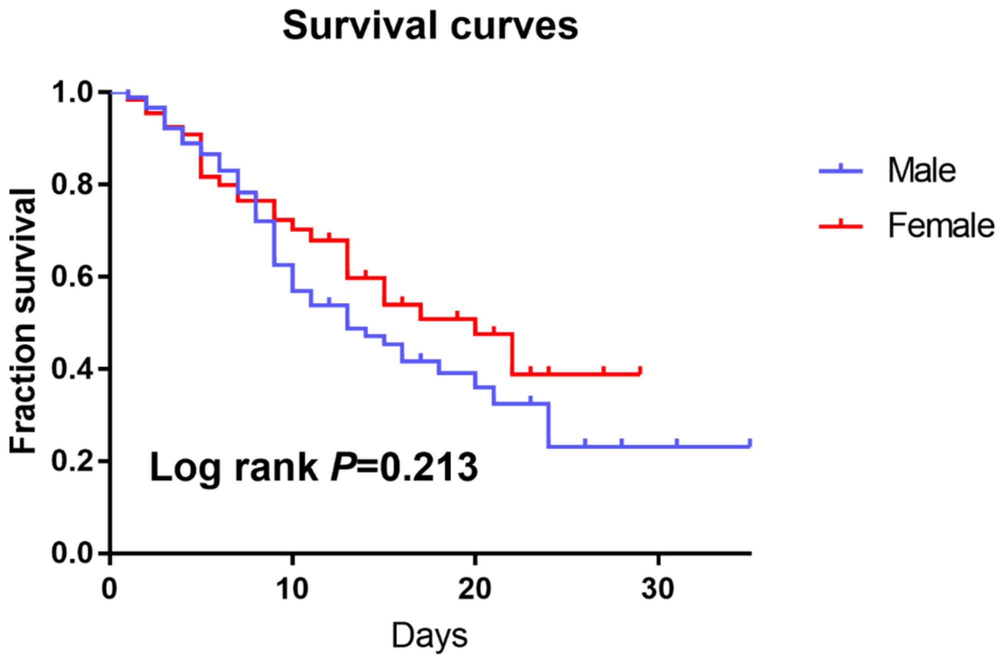

Supplementary Figure 1. Survival curves of male and female COVID-19 patients. There was no significant difference in mortality rate in ICU between male and female patients.

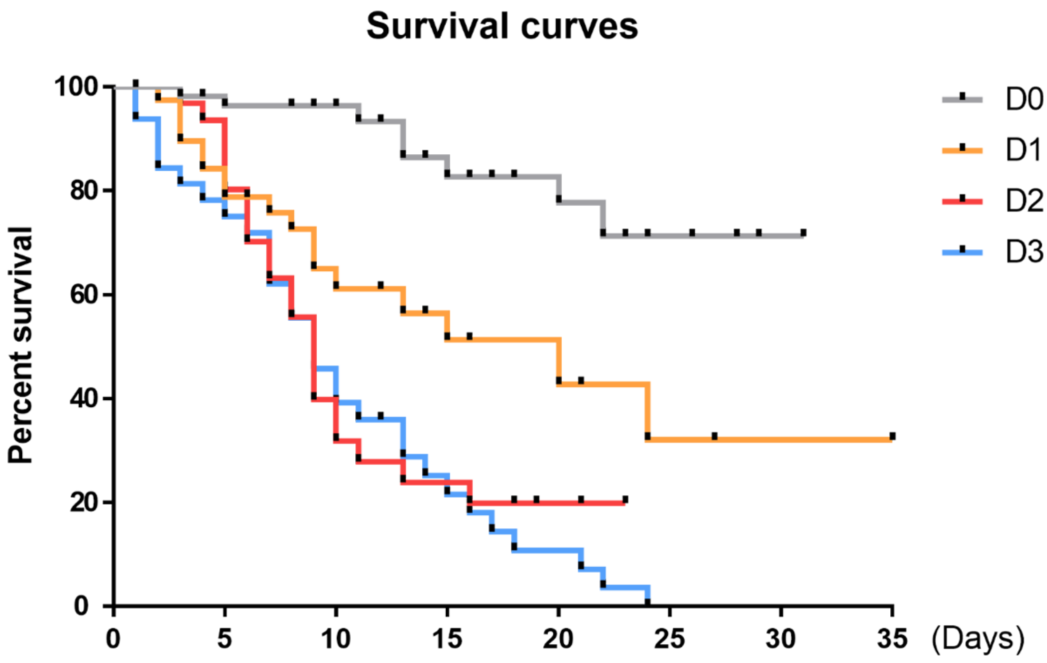

Supplementary Figure 2. Survival curve of the final mortality. D0: D-dimer<1.5μg/mL, D1: 1.5≤D-dimer<10μg/mL, D2: 10≤D-dimer<40μg/mL, D3: D-dimer≥40μg/mL.
