# Supplementary material for: Association of D-dimer elevation with inflammation and organ dysfunction in ICU patients with COVID-19 in Wuhan, China: a retrospective observational study
Source: Aging (Albany NY). 2021 Feb 11;13(4):4794–810. doi: 10.18632/aging.202496 (PMC7950237; doi:10.18632/aging.202496)
Supplement: Supplementary Tables [file aging-13-202496-s002.pdf]

SUPPLEMENTARY TABLES

Supplementary Table 1. Spearman's rank correlation analysis for the association between D-dimer and other clinical characteristics.

	R	R2	P
Age	0.166	0.028	**0.037**
Gender, Male	-0.206	0.042	**0.009**
Anticoagulant therapy	0.081	0.007	0.312
WBC (×109/L)	**0.523**	0.274	**<0.001**
Lymphocyte(×109/L)	**-0.419**	0.176	**<0.001**
Neutrophil (×109/L)	**0.557**	0.310	**<0.001**
Monocyte (×109/L)	-0.010	0.000	0.898
RBC (×1012/L)	-0.069	0.005	0.391
HGB (g/L)	-0.031	0.001	0.701
PLT (×109/L)	-0.289	0.084	**<0.001**
PFDP (μg/mL)	0.910	0.828	**<0.001**
Fbg (g/L)	-0.154	0.024	0.056
TT (s)	0.081	0.007	0.315
PT (s)	0.390	0.152	**<0.001**
PTA (%)	-0.377	0.142	**<0.001**
APTT (s)	0.001	0.000	0.990
AT3 (%)	-0.129	0.017	0.140
CRP (>160 mg/L)	**0.441**	0.194	**<0.001**
PCT (>0.5 ng/mL)	**0.357**	0.127	**<0.001**
Serum ferritin (>2000 ng/mL)	0.265	0.070	**<0.001**
Cholesterol (mmol/L)	-0.219	0.048	**0.006**
Triglyceride (mmol/L)	0.281	0.079	**<0.001**
Apolipoprotein A-1 (g/L)	-0.343	0.118	**<0.001**
Apolipoprotein B (g/L)	0.059	0.003	0.479
Lipoprotein a (mg/L)	-0.085	0.007	0.306
HDL (mmol/L)	-0.384	0.147	**<0.001**
LDL (mmol/L)	-0.210	0.044	**<0.001**
hnTNI (pg/mL)	**0.562**	0.316	**<0.001**
BNP (pg/mL)	0.426	0.181	**<0.001**
LDH (U/L)	**0.633**	0.401	**<0.001**
HBDH	**0.654**	0.428	**<0.001**
CK (U/L)	0.135	0.018	0.094
CK-MB (U/L)	0.393	0.154	**<0.001**
ALB (g/L)	**-0.524**	0.275	**<0.001**
ALT (U/L)	0.227	0.052	**0.004**
AST (U/L)	0.332	0.110	**<0.001**
Cholinesterase (U/L)	-0.415	0.172	**<0.001**
Cr (μmol/L)	0.162	0.026	**0.044**
eGFR (ml/(min*1.73m2))	-0.131	0.017	0.112
Uric acid (umol/L)	-0.107	0.011	0.181
Cystatin-c (mg/L)	0.265	0.070	**0.001**
DIC (ISTH criteria)	0.314	0.099	**<0.001**
PaO2/FiO2<100	0.027	0.001	0.738

Supplementary Table 2. Univariate and multivariate Cox regression analysis for the outcome of COVID-19 patients.

	Univariable		Multivariable	
	HR	P	HR	P
Age	1.037 (1.016-1.058)	<0.001	1.057 (1.028-1.086)	<0.001
Gender (vs male)	0.570 (0.471-1.194)	0.225		
Sofa score	1.468 (1.313-1.641)	<0.001	1.191 (1.011-1.403)	0.037
hsTNI				
≤ 28	1 (ref)			
>28	4.996 (2.963-8.426)	<0.001		
D-dimer				
<1.5	1 (ref)		1 (ref)	
1.5-10	4.202 (1.811-9.751)	0.001	3.600 (1.455-8.911)	0.006
10-40	8.335 (3.673-18.910)	<0.001	4.160 (1.727-10.022)	0.001
>40	10.016 (4.570-21.950)	<0.001	2.732 (1.077-6.927)	0.034
WBC	1.052 (1.025-1.080)	<0.001		
LYM	0.340 (0.170-0.680)	0.002		
IL-6	1.005 (0.996-1.015)	0.241		
PCT				
<0.05	1 (ref)			
0.05-0.5	3.447 (1.519-7.823)	0.003		
>0.5	9.716 (4.300-21.950)	<0.001		
Serum ferritin				
<1000	1 (ref)			
1000-2000	2.000 (1.064-3.760)	0.031		
>2000	1.897 (1.051-3.422)	0.034		
Cr				
≤133	1 (ref)			
>133	1.692 (0.891-3.215)	0.108		
CRP				
≤160	1 (ref)			
>160	2.686 (1.695-4.254)	<0.001		
ALT	1.0001 (0.999-1.003)	0.157		
LDH, per 10 increase	1.013 (1.009-1.017)	<0.001	1.010 (1.003-1.017)	0.005
CHOL	0.729 (0.567-0.936)	0.013		
TC	1.000 (0.998-1.002)	0.865		
HDL	0.210 (0.082-0.540)	0.001		
LDL	0.853 (0.574-1.268)	0.432		
CHE, per 100 increase	0.980 (0.968-0.991)	0.001		
APOA	0.075 (0.023-.250)	<0.001	0.148 (0.039-0.554)	0.005
APOB	0.945 (0.919-0.972)	<0.001	0.951 (0.924-0.978)	<0.001

Supplementary Table 3. Abbreviation and range of normal values of laboratory results.

Abbreviation	laboratory chemistries	range of normal values	unit
D-dimer	D-dimer	0-1.5	ug/mL
Fbg	Fibrinogen	2-4	g/L
TT	Thromboplastin time	13-21	s
PT	Prothrombin time	75-125	%
PTA	PT activity	10.5-13.5	s
FDP	Fibrin degradation product	0-5	ug/mL
AT3	Antithrombin III	75-125	%
INR	International Normalized Ratio	0.8-1.2	-
APTT	Activated partial TT	21-37	s
IL-6	Interleukin-6	0-7	pg/mL
PCT	Procalcitonin	<0.5ng/ml	ng/mL
ESR	Erythrocyte Sedimentation Rate	0-20	mm/h
Ferritin	Ferritin	21.8-274.66	ng/mL
CRP	C-reactive protein	0-6	mg/L
BNP	B-type natriuretic peptide	0-100	pg/mL
hsTnI	Hypersensitive sensitive troponin I	0-28	pg/mL
Myoglobin	Myoglobin	0.0-146.9	ng/mL
CK	Creatine kinase	50-310	U/L
CK-MB	Creatine kinase-MB	0-24	U/L
AMY	Amylase	35-135	U/L
LPS	Lipase	8-78	U/L
CHE	Cholinesterase	5000-12000	U/L
DBIL	Direct Bilirubin	0-8	umol/L
IBIL	Indirect bilirubin	0-13	μmol/L
GLOB	Globulin	20-40	g/L
ALB	Albumin	40-55	g/L
ALT	Alanine aminotransferase	7-40	U/L
AST	Aspartate aminotransferase	13-35	U/L
TBIL	Totalbilirubin	0-21	μmol/L
CREA	Creatinine	57-111	umol/L
UREA	Urea	3.1-9.5	mmol/L
eGFR	estimated Glomerularfiltrationrate	>90	ml/(min*1.73M^2)
Ca	Calcium	2.11-2.52	mmol/L
Cl	Chloridion	96-108	mmol/L
K	Kalium	3.5-5.3	mmol/L
Na	Sodion	137-147	mmol/L
ALP	Alkaline phosphatase	50-135	U/L
CHOL	Cholesterol	3.3-5.2	mmol/L
GGT	Gamma-glutamyl transpeptidase	7-45	U/L
UA	Uric acid	208-428	umol/L
GLU	Glucose	3.9-6.1	mmol/L
5-NT	5'-nucleotidase	0-10	U/L
AFU-L	alpha-L-fucosidase	0-40	U/L
APOA	Apolipoprotein A	1.0-1.60	g/L
APOB	Apolipoprotein B	0.6-1.1	g/L
CYSC	Cystatin C	0.6-1.55	mg/L
HDL	High density lipoprotein	1.16-1.42	mmol/L
LDL	Low density lipoprotein	2.1-3.37	mmol/L
LPa	Lipoprotein a	0-300	mg/L
PA	Prealbumin	180-350	mg/L
RBP	Retinol binding protein	25-70	ug/mL
SAA	Serum amyloid protein	0-10	mg/L
TBA	Total bile acid	0-12	umol/L
TG	Triglycerides	0.51-1.70	mmol/L

aHBDH	α-Hydroxybutyrate dehydrogenase	72-182	U/L
LDH	Lactate dehydrogenase	120-250	U/L
BAS#	Basophilic granulocyte (absolute value)	0-0.06	10^9/L
BAS%	Basophilic granulocyte (percentage)	0-1	%
EOS#	Eosinophilic granulocyte (absolute value)	0.02-0.52	10^9/L
EOS%	Eosinophilic granulocyte (percentage)	0.4-8.0	%
HCT	Hematokrit	35-45	%
HGB	Hemoglobin	115-150	g/L
LYM#	Lymphocyte (absolute value)	1.1-3.2	10^9/L
LYM%	Lymphocyte (percentage)	20-50	%
MCH	Mean corpuscular hemoglobin	27-34	pg
MCHC	Mean corpusular hemoglobin concerntration	316-354	g/L
MCV	Mean corpuscular volume	82-100	fL
MON#	Monocyte (absolute value)	0.1-0.6	10^9/L
MON%	Monocyte (percentage)	3-10	%
MPV	Mean platelet volume	9-13	fL
NEU#	Neutrophile granulocyte (absolute value)	1.8-6.3	10^9/L
NEU%	Neutrophile granulocyte (percentage)	40-75	%
PDW	Platelet distribution width	9-17	%
PLT	Platelets	125-350	10^9/L
RBC	Red blood cell	3.8-5.1	10^12/L
RDW-CV	Red cell distribution width-coefficient of variation	11-16	%
RDW-SD	Red cell distribution width-Standard deviation	37-50	fL
PLTCT	Thrombocytocrit	0.17-0.35	-
WBC	White blood cell	3.5-9.5	10^9/L
